# Patterns of Physical Activity and the Risk of Coronary Heart Disease: A Pilot Study

**DOI:** 10.3390/ijerph15040778

**Published:** 2018-04-17

**Authors:** Mustafa Al-Zoughool, Haila Al-Ahmari, Altaf Khan

**Affiliations:** 1 Department of Community and Environmental Health, King Saud Bin Abdulaziz University for Health Sciences, Riyadh 11426, Saudi Arabia; ALAHMARIH002@ksau-hs.edu.sa; 2 King Abdulla International Medical Research Center, King Saud Bin Abdulaziz University for Health Sciences, Riyadh 11426, Saudi Arabia; 3 Biostatistics Section, King Abdulla International Medical Research Center, King Saud Bin Abdulaziz University for Health Sciences, Riyadh 11426, Saudi Arabia; Khanal@ngha.med.sa

**Keywords:** sedentary behavior, cardiovascular diseases, occupational physical activity, walking

## Abstract

*Background*: In the current study, we investigated the effect of physical activity (PA) on the risk of coronary heart disease (CHD). *Methods*: In total, 146 cases of CHD and 157 matched controls were included in the study. Data on sociodemographics, lifestyle, and medical history factors were collected using an interviewer-administered questionnaire. A standard World Health Organization (WHO)-based lifestyle questionnaire was used to assess PA. The risk of CHD was analyzed in relation to PA patterns using logistic regression. *Results*: Vigorous-intensity leisure PA was not associated with a lower risk of CHD. Subjects in the highest tertile of moderate occupational PA had a significantly lower risk of CHD compared to the lowest tertile (adjusted odds ratio (OR) 0.31, 95% confidence intervals (CI) 0.17–0.56). Subjects in the highest tertile of walking hasd an adjusted OR of 0.37 (95% CI 0.20–0.70). Subjects in the medium and highest tertiles of sedentary behavior had adjusted ORs of 2.01 (95% CI 1.06–3.79) and 3.88 (95% CI 2.14–7.02), respectively (*p*-value for trend < 0.001). *Conclusion*: The current results showed that both moderate occupational PA and walking protected against CHD. Sedentary behavior increased the risk of CHD.

## 1. Introduction

Cardiovascular disease (CVD) is the leading cause of mortality and morbidity worldwide, measured by disability-adjusted life years [1,2]. According to the Global Burden of Disease Study 2013, global all-age mortality from CVD was estimated to be more than 17.2 million [1]. The World Health Organization (WHO) estimated that 31% of all deaths worldwide can be attributed to CVD, and more than 75% of these deaths occur in low- and middle-income countries [3]. In 1990, CVD ranked as fourth among leading causes of mortality worldwide but first in 2013 [1], indicating that the burden of the disease is increasing.

In the Arab world, ischemic heart disease was the leading cause of mortality in 2010, contributing to 14.3% of all deaths [4]. Over the past few decades, Arabi Gulf countries have experienced rapid socioeconomic changes characterized by large-scale urbanization, westernization, and increased individual wealth and prosperity [5]. This has been reflected in significant lifestyle changes, including increased consumption of fast and processed food, complete reliance on motorized transportation, and widespread ownership of appliances and goods that promote sedentary behavior, such as TVs, computers, and electronic gadgets [5]. Consequently, the epidemiological profile of CVD in these countries has become very similar to that in Western Europe and North America [4].

Numerous studies have shown a strong positive association between lifestyle factors—such as unhealthy dietary pattern [6,7], insufficient physical activity (PA) [8,9,10], and smoking [11,12,13,14]—and the risk of CVD. These risk factors are modifiable, suggesting that behavior modification can reduce the risk of CVD. Previous studies showed that PA may not always provide protective effect against the risk of CVD. For example, the negative association between PA and cumulative CVD risk has been observed only in certain age groups, suggesting the interaction of PA with other factors, such as stress, gender, or the environment [15]. Other studies have shown that intensive household [16] or work-related PA [17] may not reduce the risk of CVD. Examination of different patterns and intensities of PA is crucial to determine which particular type of activity may reduce the risk of CVD. Furthermore, understanding the interacting and confounding effects of other lifestyle risk factors, such as dietary patterns and smoking, may reveal some caveats regarding the association between PA and the risk of CVD.

A few studies have been conducted in the Arab world [18,19,20] or in Saudi Arabia, in particular [21,22,23], on the risk of CVD. These studies reported a high prevalence of physical inactivity that ranged from 37% in Syria [19] to 73% among Saudi women [23]. In the later study, the prevalence of physical inactivity increased with age. The aim of the present study is to investigate the prevalence and effects of PA and sedentary behavior on the risk of CVD in Saudi Arabia. Furthermore, we examined potential interaction between PA and gender on the risk of and CVD.

## 2. Materials and Methods

### 2.1. Study Population

Our study is an ongoing cross-sectional study to investigate the prevalence and effect of lifestyle factors (mainly PA) on the risk of CVD. The study also examines other risk factors such as dietary pattern, prevalence of exposure to outdoor and indoor air pollution, and smoking. In the current analyses, participants include cases of coronary heart disease (CHD) and control subjects from Prince Sultan Medical Center, also known as Riyadh Armed Forces Hospital, located in Riyadh City, Saudi Arabia. Cases of CHD were defined as patients who were diagnosed with the disease in the past year. Eligible cases were identified among those admitted to the coronary care unit with a doctor’s diagnosis confirmed by a cardiologist based on clinical presentation and laboratory examinations with any of the following: ST/non-ST elevation myocardial infarction, stable/unstable angina, or heart failure. Initially, we identified 207 cases of CVD. Several cases either refused to participate or were excluded for a variety of reasons, including being too sick to complete the questionnaires, living outside Saudi Arabia, or having been diagnosed with the disease more than one-year ago. At least one control for each case was recruited from the same hospital, matched by age (±5 years) and gender. The controls have never been diagnosed with CVD and were admitted for reasons other than diabetes, hypertension, dyslipidemia, and respiratory problems, and were not obese or overweight. Controls were recruited from non-CVD-related clinics, such as ear, nose and throat (ENT), orthopedic, and radiology, and were selected based on the matching criteria of gender and age. In total, 235 subjects were approached or recruited as controls. Exclusion was based on several criteria, including obesity. Some of the controls that refused to participate indicated that the main reason was that they did not have time. The remaining control subjects showed no interest in participating in the study.

Ethical approval was obtained from the Internal Review Board of King Abdulla International Medical Research Centre, King Saud Bin Saud University for Health Sciences (grant SP15-086). Each participant provided written informed consent.

### 2.2. Data Collection

#### 2.2.1. Demographic Data

Trained researchers administered the structured questionnaire in a uniform and standardized manner. The questionnaire included questions about demographic variables (age, sex, socioeconomic status, and education) and family history of CVD, diabetes mellitus, hypertension, and hyperlipidemia.

#### 2.2.2. Measurement of Physical Activity

To assess PA, we used the Arabic version of the WHO Global Physical Activity Questionnaire (GPAQ) for PA surveillance (http://www.who.int/chp/steps/GPAQ/en/). The part of the survey on PA contained questions about average frequency (days/week) and duration (hours/day) of moderate- and vigorous-intensity occupational PA and leisure time PA, along with frequency and duration of walking. According to the questionnaire, “vigorous-intensity activities” are activities that require hard physical effort and cause large increases in breathing or heart rate, whereas “moderate-intensity activities” are those that require moderate physical effort and cause small increases in breathing or heart rate. Subjects were also asked about the number of hours per day spent in sedentary behavior apart from sleeping (e.g., watching TV and using computers and cellphones). Hours per week of moderate- and vigorous-intensity PA and walking were converted to metabolic equivalents (METs)/week based on the following values: 3.6 METs for moderate PA and walking and 7.1 METs for vigorous-intensity PA. Total METs/week for each participant were computed by adding all METs for each type of PA. Values for METs were derived from the studies of Gong et al. (2013) [24] and Jette et al. (1990) [25]. Variables for the number of hours/week of moderate- and vigorous-intensity PA were created for activities carried out at work and during leisure time. Two other variables were also created for number of hours/week of walking and number of hours/day of sedentary behavior.

#### 2.2.3. Smoking, Body Mass Index, and Dietary Patterns

Smoking status was evaluated according to history of cigarette and waterpipe smoking (current smoker, nonsmoker, and ex-smoker). Information on the number of years of smoking and packs per week was also obtained from the subjects. In addition, exposure to environmental tobacco smoke at home and at work was evaluated (number of persons smoking, average hours spent with smokers, and number of years living with smokers). Weight and height measurements were taken to obtain body mass index (BMI). Dietary pattern was assessed using the dietary assessment section of the WHO STEPwise approach to surveillance of noncommunicable diseases (the STEPS Instrument, http://www.who.int/chp/steps/instrument/en/). This included questions about the frequency (number of days/week) and amount (number of servings/day) of consumption of fruits, vegetables, dairy products, meat products, seafood, and traditional diet.

### 2.3. Data Analysis

Data were analyzed using SAS statistical software version 9.4 (North Carolina State University, Cary, NC, USA). Chi-square tests and paired *t*-tests were used to compare proportions and means, respectively, of important variables between cases and controls. The results with *p*-values equal to or less than 0.05 were considered statistically significant. Parametric univariate and multivariate models with conditional logistic regression analyses were used to determine the association between different variables and the risk of CHD. Odds ratios (ORs) and 95% confidence intervals (CIs) were calculated for the different variables. The multivariate logistic regression model was adjusted for age, gender, education, and smoking, as well as other variables that resulted in *p* < 0.1 in the univariate model. Continuous variables of different types of PA and dietary patterns were converted into categorical variables of tertiles and were entered into the multivariate models. The median value of the lowest tertile was used as the reference in these analyses. To further examine the relationship between sedentary behavior and the risk of CHD, we used a semi-parametric regression model with natural cubic splines. These methods are based on smooth polynomial functions to examine nonlinear relationships of risk and outcome [26]. Effect of interaction between gender and PA on the risk of CHD was examined by chi-square and then by logistic regression of the CHD risk in men and women separately.

## 3. Results

A total of 303 subjects (146 CHD cases and 157 controls) were recruited in the current study. Selected characteristics of the study participants are shown in Table 1. CHD cases were older (mean age 53.7 years) than controls (mean age 49.3 years). There was an almost equal distribution of males and females among cases and controls. Cases were more likely to have a lower education level, high cholesterol, hypertension, and diabetes compared with controls. In addition, there was higher exposure to environmental tobacco smoke at home among the cases.

Prevalence and effects of different types of PA among cases and controls are shown in Table 2. Vigorous-intensity occupational PA was associated with a non-significant increase in risk of CHD (adjusted OR 1.31, 95% CI 0.70–2.46). However, moderate occupational PA was associated with more than 60% reduction in CHD risk (adjusted OR 0.38, 95% CI 0.23–0.64). Those in the highest tertile of moderate occupational PA showed the lowest risk of CHD (adjusted OR 0.31, 95% CI 0.17–0.56) compared with the lowest tertile, followed by those in the medium tertile (adjusted OR 0.53, 95% CI 0.28–0.99, *p*-value for trend 0.007). Walking was negatively associated with CHD risk (OR 0.53, 95% CI 0.31–0.91). Subjects in the highest tertile of walking time had an adjusted OR of 0.37 (95% CI 0.20–0.70). With regard to leisure time PA, vigorous exercise was not associated with increased risk of CHD (adjusted OR 1.16, 95% CI 0.61–2.19). Participants engaging in more than 1 h/week of moderate PA (highest tertile) had a lower risk of CHD compared to those in the lowest tertile (crude OR 0.39, 95% CI 0.16–0.97), but the OR increased to 1.10 (95% CI 0.65–1.89) after adjusting for confounding variables (age, gender, education, dietary pattern, and exposure to environmental tobacco smoke). Sedentary behavior, which was measured by time participants were sedentary, was associated with an increased risk of CHD. The adjusted ORs for subjects in the medium and highest tertiles of sedentary behavior time were 2.01 (95% CI 1.06–3.79) and 3.88 (95% CI 2.14–7.02), respectively (*p*-value for trend < 0.001). Overall, PA was associated with a lower risk of CHD; subjects in the highest tertile for total METs had a lower risk of CHD (adjusted OR 0.42, 95% CI 0.22–0.79).

**Figure 1:**
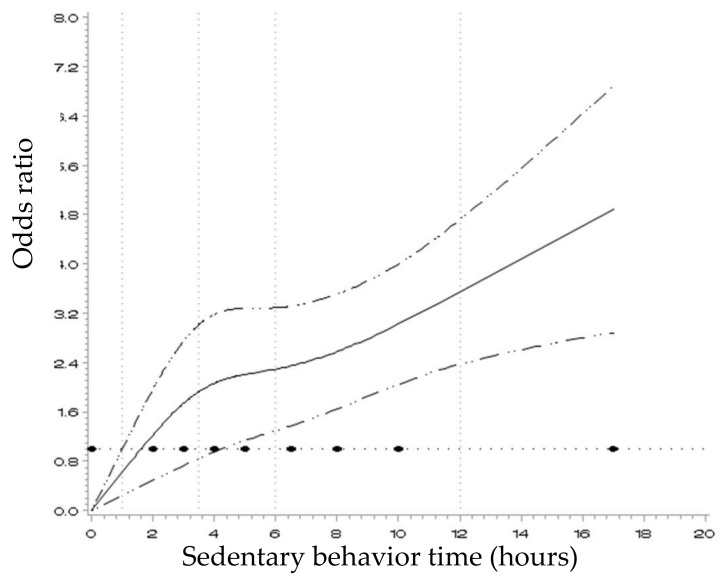
Natural cubic spline fitting of the association between sedentary behavior time and the risk of CHD. The horizontal line represents the reference value of OR = 1.0. The solid curve represents ORs for the risk of CHD and the dashed curves represent the 95% CIs.

To further explore the relationship between sedentary behavior and the risk of CHD, natural cubic splines were fitted. As shown in Figure 1, there was a nonlinear positive relationship between hours of sedentary behavior and risk of CHD.

The risk of CHD increased at a slower rate for 4–6 h of sedentary behavior time, and then increased more steeply with longer sedentary behavior time (>6 h). 

Further, we explored the interaction between intensity of PA both at work and during leisure time with other variables. PA was classified into three levels based on whether the subject engaged in vigorous- or moderate-intensity PA or was mainly sedentary (Table 3).

The results showed that gender (*p*-value 0.007) and hypertension (*p*-value 0.016) affected the levels of work-related PA to modify the risk of CHD. Among cases, there was higher proportion of hypertensive subjects (55%) who were engaged in vigorous-intensity PA than among controls (20%).

Table 4 shows the risk of CHD (OR and 95% CI) associated with work-related PA in both men and women.

Vigorous occupational PA was associated with a non-significant increase in risk of CHD in men (adjusted OR 1.74, 95% CI 0.73–4.16) and women (adjusted OR 1.14, 95% CI 0.43–3.04). By contrast, moderate PA was associated with a significantly lower CHD risk in both genders; however, this was more pronounced in men (adjusted OR, 0.36, 95% CI 0.18–0.72) than in women (adjusted OR 0.46, 95% CI 0.22–0.98). Furthermore, when moderate occupational PA was categorized into tertiles, the gender difference between CHD risk was more defined, whereas a 75% reduction in risk (adjusted OR 0.27, 95% CI 0.12–0.60) was observed in the highest tertile of moderate PA among male subjects, a non-significant lower risk (adjusted OR 0.45, 95% CI 0.18–1.10) was found among female subjects.

## 4. Discussion

In the current study, we examined the effects of engaging in PA and sedentary behavior on the risk of CHD. The results showed that moderate-intensity occupational PA as well as walking protected against CHD. Leisure PA did not have a protective effect against CHD. Our results also highlighted the strong adverse effect of sedentary behavior on the risk of CHD. This behavior is very common in the Gulf region; the prevalence was estimated to be as high as 73% among women in Saudi women, for example [23].

The rapid sociodemographic transition in the Arabian Gulf introduced substantial changes in lifestyle that was largely characterized by replacement of traditional diets with diets higher in fat and refined and processed foods, and more comfortable lifestyle that discourages PA. These changes contributed to an increased prevalence of non-communicable diseases, such as CVD, diabetes, and cancer [27,28,29,30]. In addition, the prevalence of obesity—which is an important risk factor for CVD—is alarmingly high in all Gulf countries: about 35.2% in Saudi Arabia, 42.8% in Kuwait, and 33.7% in the United Arab Emirates [31]. The obesity epidemic may be largely attributed to the increased consumption of a high-calorie, fat- and sugar-containing diet [5], along with widespread lack of PA [32].

The current finding of a low prevalence of PA was consistent with results from previous studies, which showed that Gulf countries have one of the world’s highest rates of insufficient PA; for example, 68.8% in Saudi Arabia, 64.5% in Kuwait, and 45.9% in Qatar [32]. A number of factors contribute to low prevalence of PA in the Gulf region, including increased wealth and prosperity. These resulted in significant socioeconomic changes, such as employment in professional, office-based occupations, increased use of vehicles, and a decrease in domestic activities because of affordability of maids and helpers at home [33,34,35]. In addition, hot weather during most of the year discourages outdoor activity [35].

The current finding of a lack of protective effect of vigorous-intensity PA, especially at work, is consistent with results from previous studies. A number of studies investigating the association between work-related PA and the risk of CVD reported increased risk [36,37,38,39]. For example, Cheng et al. [36] reported increased risk of acute myocardial infarction (OR 1.44, 95% CI 1.06–1.94) with heavy work-related PA. Similarly, Moe et al. [39] found that the association between metabolic syndrome and risk of death from CVD is stronger among persons with heavy work load compared with persons with much walking/light lifting. Heavy occupational PA is normally part of jobs associated with long-term fatigue and exhaustion, such as heavy lifting. Such jobs can also be stressful, thus contributing to increased CHD risk. Consistent with these studies, we found that vigorous occupational PA was associated with a non-significant increased CHD risk, and a significant interaction with hypertension. The current sample taken from a military hospital may be diverse in terms of work-related tasks that can range from sedentary to heavy work (e.g., lifting heavy machinery).

The lack of a protective effect against CHD with moderate- and vigorous-intensity PA during leisure time is puzzling considering the ample evidence from the literature of an inverse association. This may be attributed to residual confounding, the very low prevalence of this type of PA in the current population, or measurement errors. Another possible explanation is that the current population may have only engaged in leisure PA sporadically, which does not offer protection against CHD. Some studies reported that oxidative stress, which has a harmful effect on CHD, might increase with acute vigorous exercise, especially among non-athletics [40].

Moderate work PA as well as walking had protective effects, with evidence of a dose–response relationship. This is consistent with previous studies showing that moderate PA—such as walking, stair climbing, bending, or other light indoor activities—have beneficial effects on overall cardiovascular health [24,41]. Walking, in particular, is a very important type of PA, as a recent study showed that both walking and vigorous exercise are associated with substantial reductions in the incidence of cardiovascular events among postmenopausal women [42].

The current results showing a strong negative association between sedentary behavior and the risk of CHD have important implications, especially among today’s population with a low prevalence of PA [31,32]. Lack of exercise is also associated with higher risks of obesity, diabetes, and metabolic syndrome. The strong adverse effect of sedentary time, particularly of more than 5 h, necessitates action against this increasingly widespread unhealthy behavior.

The results of differential effects of moderate work PA on CHD between male and female subjects are noteworthy. The relationship was stronger for men, especially in the highest tertile. By contrast, Cheng et al. [36] found that the association was stronger in women. Nevertheless, other authors found that central obesity with the consequent metabolic syndrome, itself an important risk factor of CHD, is higher in women [43]. Additionally, blood pressure rises more steeply in aging women compared with men [44]. Other female-specific risk factors, such as hormonal dysfunction in premenopausal women, are associated with an increased risk of atherosclerosis and CHD events [45]. Other factors that interact with the association between PA and CHD in women may involve their hormonal status, such as menopausal status or use of hormone replacement therapy. However, since no information was collected in the current study about menopausal status or number of women with hormonal dysfunction, a conclusion cannot be drawn on whether the association was actually stronger in men than in women.

The current study has several limitations. Because the sample was drawn from a hospital, it might not represent a balanced distribution of the overall population with regard to risk factors. However, to reduce the effect of this selection bias, we selected controls from the same hospital. Self-reporting of most variables in the current study may introduce measurement errors as evidenced by the relatively large standard deviation of some of the PA measurements. Differential recall bias, which is common in case–control studies, is a factor that may either bias the estimates toward null or overestimate the effects of PA, depending on the extent of differential over- or under-reporting of PA by cases and controls. However, our results on total activity-related energy expenditure are consistent with those from previous studies. Thus, recall bias is less likely to play a role in our study. Another limitation is the relatively small sample size, which to some extent may limit the generalizability of the results. We are expanding the study by recruiting more subjects.

The main strength of our study is that we explored the dose–response effect of sedentary behavior, which is very common in the Gulf society. Careful examination of the association between sedentary time and CHD risk revealed a significant and rapid increased CHD risk for more than 5 h of sedentary behavior. We also collected detailed information about dietary patterns and smoking, which allowed adjustment for these important confounding variables. The important finding in this study of the possible harmful effects of vigorous-intensity occupational PA highlights the need to address this issue in occupational settings.

## 5. Conclusions

The current results showed that moderate-intensity occupational PA protected against CHD, and an increased risk of CHD was observed with sedentary behavior. Walking was also inversely associated with CHD risk. Future research should focus on understanding the specific types of PA that reduce the risk of CVD. In addition, attention must be paid to the distinct effects of occupational PA and PA during leisure time. Similarly, examining the proportion of time spent in sedentary behavior at work compared with at home is important to delineate strategies to encourage occupational PA.

It is important to implement educational programs that enhance PA at all levels, including in schools and at the workplace. Awareness should be raised of the important health benefits of sufficient PA; the WHO recommends that adults should engage in moderate-intensity aerobic PA for 300 min per week or vigorous-intensity aerobic PA for 150 min per week, or an equivalent combination of moderate- and vigorous-intensity PA.

## Figures and Tables

**Table 1:** Sociodemographic characteristics of participants.

Characteristic	Controls *N* = 157		Cases *N* = 146	*p*-Value
Age (mean ± SD)	49.4 ± 7		53.7 ± 9	0.21
Gender *n* (% female)	45		44	0.90
BMI * (kg/m^2^) *n* (%)				0.14
<25	42(27)		29(20)	
25–30	58(37)		49(34)	
>30	57(36)		68(46)	
Education *n* (%)				0.01
Illiterate	10(6)		25(17)	
Primary school	13(8)		18(13)	
Secondary	16(11)		17(12)	
High school	33(21)		34(23)	
University	71(45)		39(27)	
Graduate	14(9)		12(9)	
Income (Saudi Riyals) *n* (%)				0.18
<5000	39(19)		42(29)	
5000–9999	45(29)		44(30)	
10,000–19,000	57(36)		43(29)	
>20,000	15(16)		18(12)	
Family history of CVD *n* (%)	35(22)		45(32)	0.05
High cholesterol	35(22)		72(51)	<0.00
Hypertension	42(27)		77(54)	<0.00
Diabetes	38(24)		34(38)	0.00
Current smoker *n* (%)	16(10)		20(14)	0.26
Environmental tobacco smoke				
Exposure at home *n* (%)	38(24)		54(38)	0.01
Exposure at work *n* (%)	72(46)		68(48)	0.72
Mean (±SD) consumption of fruits				
(servings/day)	1.05 ± 0.2		0.90 ± 0.1	0.20
Mean (±SD) consumption of vegetables				
(servings/day)	1.39 ± 0.3		1.24 ± 0.4	0.26
Overall engagement with PA				
Moderate-intensity *n* (%)		227(75)		
Vigorous-intensity *n* (%)		91(30)		

* BMI: body mass index; CVD: Cardiovascular disease; PA: physical activity.

**Table 2:** Coronary heart disease risk according to different types and levels of physical activity.

Type of Physical Activity	Control (%)	Case (%)	OR (95% CI)	Adjusted Odds Ratio * (95% CI)
**Occupational PA**				
**Vigorous**:				
no	84	79	1	1
yes	16	21	1.38 (0.77–2.48)	1.31 (0.70–2.46)
Mean hours/week (SD)	1.57 (2.5)	3.38 (2.98)		
**Moderate**:				
no	35	57	1	1
yes	65	43	0.42 (0.27–0.67)	0.38 (0.23–0.64)
Mean hours/week (SD)	11.11 (7.60)	6.98 (4.34)		
**Tertiles of moderate PA (hours/week)**				
Lowest 0	35	56	1	1
Medium >0–8	24	20	0.51 (0.28–0.93)	0.53 (0.28–0.99)
Highest > 8	41	24	0.36 (0.21–0.61)	0.31 (0.17–0.56)
**Leisure-time walking**:				
no	38	48	1	1
yes	62	52	0.67 (0.43–1.06)	0.53 (0.31–0.91)
Mean hours/week (SD)	2.94 (3.23)	1.67 (1.09)		
**Tertiles of walking (hours/week)**				
Lowest	38	48	1	1
Medium >0–2.1	22	25	0.93 (0.52–1.67)	0.82 (0.43–1.55)
Highest > 2.1	40	27	0.54 (0.32–0.92)	0.37 (0.20–0.70)
**Leisure PA**				
**Vigorous**:				
no	82	81	1	1
yes	18	19	1.01 (0.57–1.81)	1.16 (0.61–2.19)
Mean hours/week (SD)	0.74 (1.12)	1.67 (1.81)		
**Moderate**:				
no	62	65	1	1
yes	38	35	0.86 (0.54–1.37)	1.02 (0.62–1.69)
Mean (SD) hours/week	1.35 (1.14)	1.93 (2.04)		
**Tertiles of moderate PA (hours/week)**				
lowest	61	65	1	1
Medium (>0–1)	12	5	1.0 (0.60–1.66)	0.66 (0.25–1.76)
Highest >1	27	30	0.39 (0.16–0.97)	1.10 (0.65–1.89)
**Sedentary behavior time**				
Mean hours/day (SD)	4.52 (2.57)	6.7 (3.61)		
**Tertiles of sedentary behavior sedentary behavior time (hours/day)**				
Lowest 0–4	58	31	1	1
Medium 4–6	21	24	2.04 (1.12–3.70)	2.01 (1.06–3.79)
Highest > 6	21	45	3.96 (2.30–6.84)	3.88 (2.14–7.02)
**Tertiles of total MET**				
Lowest 0–14	32	40	1	1
Medium 15–79	33	36	0.86 (0.49–1.49)	0.73 (0.40–1.30)
Highest > 79	35	36	0.54 (0.30–0.97)	0.42 (0.22–0.79)

* Adjusted for age, gender, education, dietary pattern, and exposure to environmental tobacco smoke.

**Table 3:** Interactions between work- and leisure-related PA and other factors related to coronary heart disease.

CHD Risk Factors	Intensity of Work-Related PA *			*p*-Interaction **	Intensity of Leisure-Related PA *			*p*-Interaction
	Sedentary	Moderate	Vigorous		Sedentary	Moderate	Vigorous	
Male gender *n* (%)				0.0071 *				
Case	39 (55)	21 (49)	20 (67)		40 (45)	22 (73)	18 (67)	0.49
Control	29 (59)	43 (52)	14 (56)		49 (54)	17 (46)	20 (71)	
Cholesterol *n* (%)				0.18				0.34
Case	40 (56)	20 (27)	13 (18)		46 (52)	18 (60)	10 (37)	
Control	13 (32)	15 (45)	5 (13)		20 (22)	6 (16)	7 (24)	
Hypertension *n* (%)				0.016 *				0.42
Case	14 (47)	25 (58)	39 (55)		47 (53)	20 (67)	12 (44)	
Control	9 (36)	23 (28)	10 (20)		30 (33)	8 (22)	4 (14)	
Current smoking *n* (%)				0.18				0.55
Case	7 (10)	6 (14)	7 (23)		13 (15)	2 (7)	5 (19)	
Control	5 (10)	7 (9)	5 (20)		10 (11)	3 (8)	2 (7)	
BMI mean (SD)				0.74				0.027
Case	30 (2.3)	30 (3.1)	30 (3.9)		31 (3.1)	29.3 (2.9)	26.5 (3.1)	
Control	27 (3.1)	29 (2.9)	30 (3.0)		28.1 (2.9)	29.3 (2.0)	28.1 (2.0)	
Fruits serving/day mean (SD)				0.91				0.1
Case	0.86 0.91)	1.02 (0.7)	0.83 (0.52)		0.83 (0.5)	1.26 (0.72)	0.76 (0.42)	
Control	0.91 (0.7)	1.16 (0.9)	1.02 (0.61)		1.17 (0.61)	0.99 (0.49)	0.73 (0.40)	
Vegetables serving/day mean (SD)				0.54				0.13
Case	1.22 (1.01)	1.32 (0.92)	1.22 (0.72)		1.18 (0.50)	1.58 (0.61)	1.11 (0.29)	
Control	1.25 (0.91)	1.39 (0.85)	1.66 (0.89)		1.48 (0.70)	1.23 (0.71)	1.31 (0.37)	

* Levels of PA of sedentary, moderate, and vigorous were chosen based on the WHO definition. Please see the methods section for more details. ** Statistically significant at *p* < 0.05.

**Table 4:** Association between work-related PA and CHD by gender.

Type of PA	Control (%)	Case (%)	*p*-Value	OR (95% CI)	Adjusted OR * (95% CI)
*Men*					
Vigorous PA			0.16		
No	84	75		1	1
Yes	16	25		1.71 (0.79–3.63)	1.74 (0.73–4.16)
Moderate			0.016		
No	36	60		1	1
Yes	64	40		0.37 (0.19–0.69)	0.36 (0.18–0.72)
Tertiles of moderate PA (hours/week)			0.007		
Lowest	36	60		1	1
Medium	15	18		0.68 (0.28–1.64)	0.63 (0.23–1.68)
Highest	49	21		0.26 (0.13–0.53)	0.27 (0.12–0.60)
*Women*	s				
Vigorous PA			0.91		
No	77	78		1	1
Yes	23	22		0.99 (0.39–2.52)	1.14 (0.43–3.04)
Moderate			0.045		
No	36	57		1	1
Yes	64	43		0.5 (0.25–0.99)	0.46 (0.22–0.98)
Tertiles of moderate PA (hours/week)			0.16		
Lowest	34	50		1	1
Medium	35	24		0.44 (0.19–0.99)	0.48 (0.20–1.16)
Highest	31	26		0.56 (0.25–1.28)	0.45 (0.18–1.10)

* Adjusted for age, gender, education, dietary pattern, and exposure to environmental tobacco smoke.
